# Exploring *proximity*-based recommendation criteria as a tool for information exchange and interactions between locals and tourists

**DOI:** 10.1007/s11042-022-13369-y

**Published:** 2022-07-08

**Authors:** Chiara Ceccarini, Valentina Nisi, Catia Prandi

**Affiliations:** 1 Department of Computer Science and Engineering, University of Bologna, Bologna, Italy; 2 Técnico, University of Lisbon, Lisbon, Portugal; 3 ITI/LARSyS, Funchal, Portugal

**Keywords:** Sharing economy, People-to-people recommendation, Proximity, Authentic tourism

## Abstract

Sharing economy and contemporary tourism are two emerging concepts that urge to be investigated together with new ubiquitous and immersive technologies, in the tourism and hospitality sector. In this rich scenario, we designed and implemented ShareCities, a platform to foster remote direct information exchange and meaningful interactions among tourists and locals. Exploiting ShareCities we here present an extended analysis on the opportunity to use people-to-people recommendation criteria based on proximity. We hence defined three criteria which drove our analysis: i) profile similarity, ii) geographical proximity, and iii) random exploration. Through an online questionnaire, we collect answers from 126 young-adult students, obtaining a general positive interest in the three criteria but also concerns in terms of privacy, trust, and feeling of disorientation.

## Introduction

It is undisputed that, in recent years, novel and ubiquitous technologies are having a significant effect on the tourism and hospitality sector [1, 6, 24]. This is true today more than ever due to the critical situation we are experiencing because of the COVID-19 pandemic [14, 39]. The pandemic has forced researchers and the industry to resort to emerging pervasive technologies (such as Virtual Reality) to reboot the tourism industry and regain consumer confidence [22, 42]. Indeed, the opportunity to explore such technologies in the hospitality and tourism sector is not new. In fact, the recent widespread diffusion of mobile devices that enables access to multiple sources of information in a ubiquitous, continuously connected fashion has changed the way we experience tourism-related services [16, 26].

In this rich ICT context, we frame our research considering two diverse but potentially linked concepts: i) sharing economy, and ii) contemporary tourism. Sharing economy is a new economic model that uses online platforms to match offer and demand, working in a peer-to-peer logic to connect who need with who has, favoring new forms of socialization; at tourist level it opens new opportunities and solutions often more valuable and more affordable than those provided by the traditional tourist market [7]. Contemporary tourism can be defined picturing a “tourist interested in the emotional dimension of the travel, on the opportunity to do real travelling experiences based on the connection with local community, and by the refusal of the standardisation and commodification of tourism experiences” seeking for a sustainable and tailored travel [7].

In this scenario, we present the ShareCities platform, an application (web and mobile) that allows current and “future” tourists to foster playful immersive connections with the local community. In doing so, we exploited a 360^∘^ virtual representation of a room created by locals that shows elements linked to her/his personality, hobbies, and interests. Moreover, the rooms will also show tourists what the owner suggests to see or do in the nearby area or the provided services (e.g., city tour). In this way, the tourists have the opportunity to reach authentic information and interact with the local community. Some past versions of the system, including some initial findings have already been presented in [9, 38].

In this paper, we take our research study a step further by presenting an analysis on three *proximity*-based people-to-people recommendation criteria. In particular, the findings from a user study conducted with 126 participants who impersonated a “future” tourist are presented, in order to deeply analyze three different criteria to provide tourists with meaningful recommendations of the locals rooms and, eventually, foster an authentic tourism experience. To add more details, this study is framed within a research framework aimed to investigate the *proximity* concept as an enabler for authentic tourism in the context of the sharing economy. Accordingly, we defined our research hypothesis as “Proximity affects the direct information exchange and interactions between locals and visitors in ShareCities”. As the first step, we conceptualize *proximity* both in terms of i) closeness or distance between two user’s profiles, and ii) geographical closeness or distance between two persons. Following such conceptualization, we investigated proximity as a mechanism to recommend locals profiles to tourists, while interacting with ShareCities. In particular, three definitions have been coined, representing three dimensions to consider in our analysis: 1. profile similarity: how much a tourist is similar to the locals in terms of common interests and how much such a dimension can affect the creation of empathy between the two persons; 2. geographical proximity: how much a tourist is physically near to locals in terms of geographical position and how much this physical closeness can have positive effects considering the *hyper-local tourism*^1^ trend; 3. random exploration: the system will recommend a random local and how much such possibility can benefit in terms of serendipity [30] and diversifying experiences [13]. Each dimension has been investigated in our evaluation through the implementation of three recommendation criteria, to exploit possible advantages and/or limitations in fostering interactions between locals and visitors in ShareCities.

The rest of the paper is organized as follow. Section 2 details background and related work in social connection between tourists and locals, and recommendation in the tourism context. Then, in Section 3, we present the ShareCities platform (web and mobile) and its functionality. In Section 4, we proposed three Locals-to-Tourists recommendation criteria. Then, the paper continues in Section 5 presenting the user study we carried out and the methodology, in Section 6 the results. Finally, we conclude with final remarks and future works.

## Related work

In this Section, we presented the main works and studies that inspired our approach. In particular, we will focus on three aspects: i) the sharing economy concept; ii) the connections and interactions between locals and tourists; and iii) the recommendation in touristic contexts.

### Sharing economy

Sharing economy can be considered an umbrella term as different scholars analyzed the concept and gave a broad set of definitions [2]. On this basis, Acquier et al. proposed an organizing framework that placed the sharing economy in the intersection between three areas: access economy, platform economy, and community-based economy [2]. Each of these three areas focused on different initiatives: access economy concerns the share of underutilized resources or skills to enhance their use; platform economy is related to a decentralized exchange between peers through digital platforms; while the community-based economy is about the coordination through forms of interaction that are non-contractual, non-hierarchical or non-monetized [2]. As a matter of fact, the sharing economy has been deeply investigated in the last decades, especially in the tourism context [12, 31], with a particular focus on Peer-to-Peer (P2P) accommodation [28] thanks to the increase of providers, such as AirBnB and Couchsurfing. In this context, this economic principle has been enabled by the evolving of technology and Web 2.0, which have influenced and eased the creation of trust between host and guests through digital connections, especially in the home exchange [10].

### Connection between visitors and locals

In recent years, the interaction and connection between locals and visitors are increasingly studied. In [32], Moyle et al. analyzed the cultural interchange between tourists and locals, exploiting two Australian islands as a case study. They interviewed 30 people from the local community or tourism stakeholders, and they showed how locals might have good motivations to interact with tourists. These motivations could be monetary or coming from the desire to deliver quality experiences. However, for the interaction to be positive, it is necessary to educate the local community about the positive effects that may come from it. Moreover, this interaction can make the tourist feel emotionally closer to the residents and, hence, impact the satisfaction and the destination loyalty, as demonstrated in [40] through surveys in Capo Verde. The intuition of analyzing the relationship between locals and tourists to investigate the effect that it could have in the destination’s image and in the intention to recommend or revisit the place was also the basis for the study of Stylidis [46].

In particular, concerning the interaction from the tourist’s perspective, the author considered the friendship with locals, the tips and recommendations about the places to visit or where to eat, the information about the city’s lifestyle, and if the interaction has improved the perceived sense of safety.

Moreover, many of the studies that analyze the relationships between travelers and locals were conducted exploiting some sharing economy providers, such as AirBnB^2^ and Couchsurfing^3^, to deeper understand the intention of tourists to live more authentic experience [15, 18, 35].

In this study, we took a step forward, investigating if proximity-based recommendation criteria can facilitate the creation of social interactions between tourists and locals before the actual meeting.

### Recommendation in tourism

To improve the touristic experience, recent works have deeply investigated the use of recommendation systems, especially in suggesting new Points of Interest (PoI) to the tourists. An example of travels’ recommendation is PhotoTrip, which suggests to tourists some unexpected and not mainstream Point of Interest [8]. In particular, it used social networks, crowdsourcing, and gamification to provide relevant photos and information of cultural heritage locations and improve the response quality of the system. In [49], Yochum et al. analyzed the Location-based recommendations in the tourism context. In particular, they found that they can be divided into two categories: the stand-alone location recommendations and the sequential location recommendations. The first group includes the suggestions for a single location after taking into account the needs and preferences of the users, their location history, or their trajectories. The second group suggests a set of locations that creates a travel route especially based on geotagged social media content or GPS trajectory. The social media photos were also exploited by Figueredo et al. in [19] in order to create a series of tourism attractions and recommend them to the user. Additionally, they used Convolutional Neural Network and fuzzy logic to extrapolate the scene profile from the photo and create the tourist profile based on the preferences to execute the recommendation. The effectiveness of the system was evaluated against ground truth and showed that 90% of the attractions provided could be considered relevant for each validation users’ profiles.

Recently, some studies have investigated the benefits of serendipity in the recommendation systems also in the tourism domain. In [30], Menk et al. aimed to surprise the users with serendipitous recommendations of places exploiting their degree of curiosity and education. In their study, the authors extracted information from social networks (e.g. Facebook app) to predict curiosity and choose the most suitable places. Finally, they evaluated the recommendation system with users, asking them to rate each suggested place using 3 questions (5-point Likert scale) to measure the level of accuracy, serendipity, novelty and textual feedback to rate the users’ satisfaction. From the evaluation, they demonstrated how the system was able to provide novel, surprising or serendipitous recommendations with a good level of accuracy.

To summarize, previous works on recommendations in the tourism domain focused on suggesting new and possibly unexpected places to tourists, often exploiting their social media presence and preferences. In our case study, we decided to change the approach and focus on recommending people, and in particular, locals. Hence, we wanted to suggest three locals’ profiles: (i) the one that best suited each tourist in terms of similarity of interests; (ii) the nearest one in terms of geographic proximity; and (iii) a random one to further investigate the concept of serendipity. It is worth to notice that, people-to-people recommendation criteria have been deeply investigated in different contexts then tourism, in particular in dating apps [27, 37] and social media networks [23, 48].

## ShareCities

The ShareCities platform aims to create a connection between a tourist visiting or planning to visit a city and one or more residents. In doing so, we exploited their personality and interests, taking advantage of the similarity and compatibility between them. Through this compatibility, we wanted to create an affinity and, potentially, empathy to make the touristic experience more authentic. The current platform is based on our previous works (details can be found in [9, 38]) and allows locals to present themselves using a virtual representation of a hypothetical room. The main idea is to use the metaphor of a room to present themselves, their interests, and their personality. The room is, in fact, customizable to include personal photos, posters, and objects. The future tourist can visualize the local’s room and start gaining information about the local’s personality and interests, and the provided services (such as city tour).

**Fig. 1:**
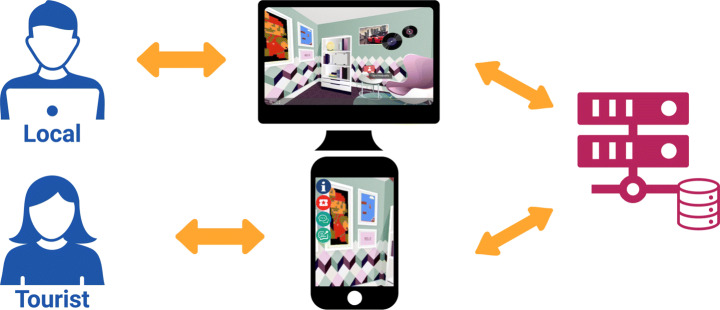
ShareCities architecture: it is a client-side architecture where the server gets the data from the database and communicates with the clients. We have two applications: one web-based, which targets the locals, and the other mobile-based, which targets the tourists

The actual platform is composed of i) a web application targeting the locals, and ii) a mobile application that targets tourists, as presented in Fig. 1. To add a few details, the web application was developed with the framework Flutter^4^ to create an app that worked well both on Android and iOS. Instead, the web application was implemented with the Javascript framework Vue.js^5^. Both of these applications communicate with a server built in Node.js,^6^ that is connected to MongoDB database^7^ that handle all the data. More details about the main functions of the two applications are presented as follows.

### Web application

**Fig. 2:**
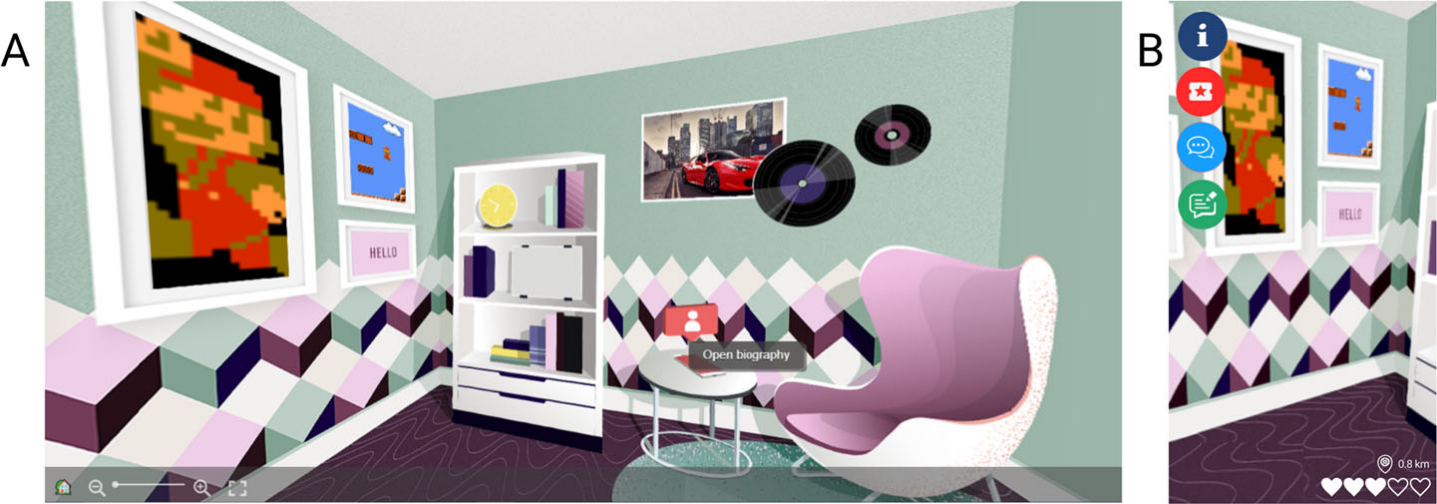
Example of a local’s room: as we can see from the room, its owner likes video games (like Super Mario), music, and cars. Moreover, with a simple click on the phone icon on the table, the local can update his/her biography, contacts, and services provided to tourists. The same room is explorable both from **(A)** the web-based app, and **(B)** the mobile app

Through the web application, locals can customized their virtual room. Each locals’ room is a 360^∘^ panoramic view of a virtual space that expresses the owner’s personality. The locals can upload images into the paintings inside the room to personalize it and show their interests. In the current version, these operations on the paintings are possible through the use of OpenCV^8^, an open-source library for computer vision and machine learning. The main operations are the following:

- insertion and removal of a picture: the local can choose a painting where to put the desired picture. Then, the image is uploaded in the room and can be seen in perspective in a 360^∘^ view. This is possible through a geometric transformation on the image before inserting it in the picture;
- insertion and removal of a picture with mask: in the room, some paintings are partially covered by another object (i.e., the vinyl that covers the left angles of a painting in Fig. 2A). Hence, to insert the new image inside of these paintings, we exploited a mechanism based on a binary mask, created with a binary AND followed by an OR between images, that identified the correct area where to put the new image that remains behind the foreground object.

Therefore, the local owner of the room can interact directly not only with the paintings but also with other objects inside, such as the phone on the table in Fig. 2A, to modify information, contacts, and services provided to tourists (e.g., city tour, culinary experience).

### Mobile application

Through the mobile application, tourists can access the different cities available on ShareCities and interact with the digital rooms employing a 360^∘^ VR view. The navigation is possible both using the fingers to explore the room, and moving the phone, exploit the sensor of the device, especially the gyroscope, and the 360^∘^ VR panorama feature to create an immersive virtual experience of the host’s room through the rotation of the device in the real world. For the 360^∘^ viewer, we used the Flutter Panorama plugin^9^. Through the exploration of the room, the tourists can create a mental image of the local’s personality and, eventually, cultivate a sense of empathy.

In addition, the information provided by the locals is visible to the tourist in their virtual room and enjoyable through ShareCities mobile app thanks to a lateral menu with a series of icons, as shown in Fig. 2B. Thanks to the menu, the tourists can see the general information (name, interests, and contacts) of the owner, the services provided, they can leave a message asking for suggestions (e.g., place to visit or where to eat), or they can write a review whether they enjoyed the services.

## Locals-to-Tourists recommendation criteria

In this paper, we focus on the investigation of different people-to-people recommendation criteria in order to understand if there is a criterion that best suites our goal: increase the likelihood of a positive interaction.

Our investigation started with a preliminary users’ study, where we asked users if they would like to have locals-to-tourists recommendation criteria (results are presented in Section 4.1). Then, we implemented the three recommendation criteria in our application, as presented in Section 4.2, and finally we performed a specific evaluation engaging more than 100 users (as detailed in Section 5).

### Preliminary user study

To collect their insights, we performed a preliminary user study engaging 19 future tourists (11 males and 8 females), recruited via the Interactive Technologies Institute (Funchal, Madeira, Portugal) mailing list. Participation was voluntary based on an informed consent. The age ranged from 24 to 42. The participants’ background was variegated, from computer science and engineering, to designer, psychology, and management. Regarding nationality, all the participants were Portuguese. We engaged one participant per session. The session was comprised of four different moments: introduction, app interaction, questionnaire, and interview. The experiment mainly focused on understanding the visitor point of view, and in particular, if the use of 360^∘^ VR personalized rooms can facilitate the creation of connections, affinity, and empathy between the tourist and the local. Details about the evaluation methodology, the participant demographic and the obtained results can be found in [38]. In addition to the main objective of the experiment, we included additional questions in the provided questionnaire, to perform a preliminary inquiry about the possibility to exploit recommendation criteria to match locals with tourists and facilitate the interaction among them.

In particular, we asked participants: 1. To what extent do you like the possibility to visualize a proximity rank (i.e., a number, from 1 to 5, inside a heart icon) that compares your profile with the local’s one?; 2. To what extent do you find useful the possibility to visualize a proximity rank (i.e., a number - from 1 to 5 - inside a heart icon) that compares your profile with the local’s one?; 3. To what extent do you think a recommendation strategy (based on the profile proximity) could benefit your experience?; 4. To what extent would you like to provide personal information to have a more accurate recommendation mechanism? (e.g., preferred color, animal, music); 5. Once in the touristic city, would you prefer to visualize the locals’ rooms order by geographical proximity (the first is the one “physical” closer to you) instead of the “profile” proximity (the first is the more similar to your profile)?.

Considering items 1., 2., and 3. the answers reveal a positive interest (with the majority of people rating 4 out of 5 such a possibility, and none selecting negative values - that are 1 or 2) with no negative answers. Item 4. obtained a more controversial outcome, with two users voting 5 (strong agree), eight voting four (agree), and eight voting three (neutral), while one voting 1 (disagree). Finally, the strong majority of people (16 out of 19) select “both profile geographical proximity” to item 5, while the remaining selected “Only profile proximity” (the option we here call profile similarity).

Speculating on the collect results and elaborated insights, we design this study.

### Recommendation criteria implementation

Inspired by the resulting insights, we included in ShareCities the possibility for a tourist to visualize locals rooms ordered by three recommendation criteria:

1. profile similarity: how much a tourist is similar to the locals in terms of common interests;
2. geographical proximity: how much a tourist is physically near to locals in terms of geographical position;
3. random exploration: the rooms will be presented in a total random order.

For the purpose of this study, we decide to provide only the best match for each criteria, to better investigate the three options.

#### Profile similarity

**Fig. 3:**
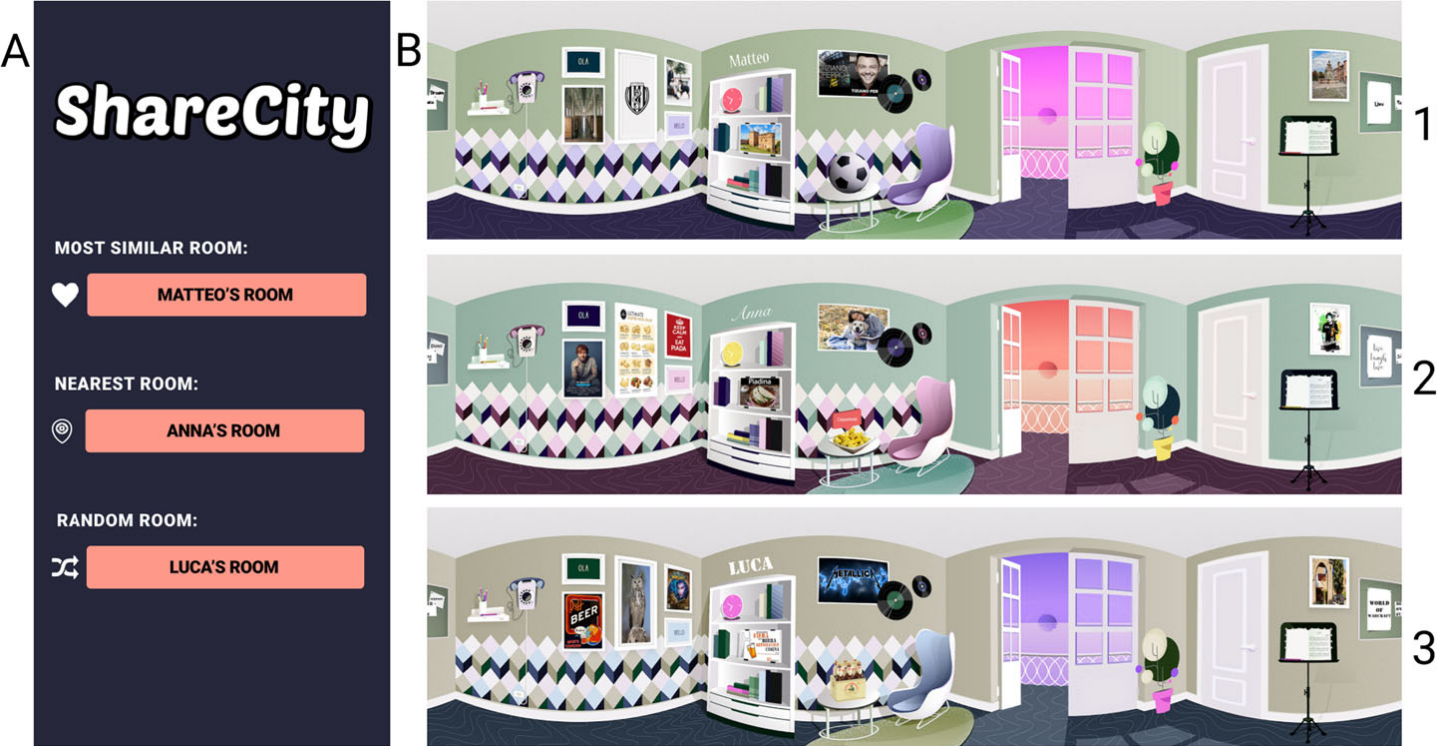
**(A)** The user interface that proposes to a tourist three different kind of locals’ profiles, in order: (i) the local most similar to the tourist (in terms of interests); (ii) the local nearest to his/her geographical position; and (iii) a random local registered into the system. **(B)** The three rooms of Matteo (1), Anna (2), and Luca (3) with all the paintings customized based on the interests and hobbies of the owner

The first room suggested to the tourist belongs to the most similar local (Fig. 3). To compute a similarity between the two individuals, we exploited the common interests. In particular, we asked the locals to choose their interests from a checklist during the creation of the room. The checklist was a shortlist of 32 popular and common hobbies in 2021.^10^ We made the same request to tourists at the time of registration. After the registration or login, the tourist can choose the city to visit, and then the interests of the tourist and locals are analyzed. As a matter of fact, we computed the similarity between the tourist and each of the locals according to the Jaccard similarity:

1 $$ Jaccard(T,L) = \frac{\textsf{T} \cap \textsf{L}}{\textsf{T} \cup \textsf{L}} $$

where *T* is the set of interests of the tourist, while *L* is the set of interest of the local. Then, as result, we chose the local with the higher Jaccard similarity score. We decided to exploit Jaccard Similarity as it is used to measure similarities between sets when categorical data or keywords are examined [34]. This method allowed tourists to discover people with similar tastes and bring them closer together and, eventually, create empathy between them and live more authentic experiences during the travel. The score for the profile similarity is also visible inside the locals’ room. We exploited five hearts icons that are colored based on the score in the left corner of the room, as shown in Fig. 2B. The profile similarity could benefit the travel of the tourists both before the actual departure (e.g., they can decide the places to visit after asking suggestions to the most similar local) and during the trip (e.g., taking advantage of the services and experiences offered or proposed by the most similar local).

#### Geographical similarity

The second room suggested to the tourist belongs to the nearest local in terms of geographical position (Fig. 3). This criterion exploited the GPS of the tourist’s mobile device in order to get the local nearest to him/her. We asked the locals, during the creation of the room, to also provide an approximate location. After the registration or login, the tourist can choose the city to visit, and then, if the GPS is on, we computed the distance between the two locations.

We computed the distance between the two geographic points exploiting the *haversine formula*, which calculates the great-circle distance between two coordinates as the shortest distance over the earth’s surface, with the following formula:

2 $$ \begin{array}{@{}rcl@{}} a &=& \sin^{2} \left( \frac{\Delta lat}{2}\right) + \cos(lat_{1}) * \cos(lat_{2}) * \sin^{2}\left( \frac{\Delta long}{2}\right)\\ d &=& 2 * R * \arcsin(\sqrt{a}) \end{array} $$

where *lat*_1_ and *lat*_2_ are the latitude of the two coordinates, Δ*lat* is the difference between *lat*_1_ and *lat*_2_, Δ*long* is the difference between the longitude of the two coordinates and R is the radius of the earth (= 6,371km). In choosing this particular formula, we were inspired by previous works [17, 41].

To provide the actual geographical distance to the tourist, we chose to display the value in kilometers in the bottom-right corner of the room (next to a marker icon and above the hears icons), as shown in Fig. 2B. This similarity could be an appropriate choice especially to avoid taking transportation or in case the tourist has little time but has the desire to live authentic experiences on site. However, contrary to profile similarity, in order to be effective, it should be used only during the trip and not before as it exploits GPS position.

#### Random exploration

The third and last room suggested to the tourist belongs to a random local registered into our system (Fig. 3). In this scenario, we proposed to the tourist a randomly chosen local, different from the most similar and the nearest. The random exploration could be a way to discover interesting experiences away from the tourist’s usual interests, providing a new approach that had not been thought of to interact with the locals.

## User Study evaluation

To evaluate the ShareCities platform, we conducted an online survey (using Google Form^11^ and Figma^12^), consisting of the two phases: testing the prototype and answering a questionnaire. We opted for an online survey due to the COVID restrictions we are still experiencing. Via the provided URL, participants were able to access the survey and read details about the research project and about data storage and usage (in accordance with the European General Data Protection Regulation). Then, they needed to give their consent to participate to the study.

### Testing the prototype

The first phase of the user study was the prototype test of the ShareCities mobile app. To easily perform this task without an additional burden (such as requiring the respondent to install the app), we replicated our working mobile app in Figma. The result prototype had the same look and feel of our mobile app.

In order to provide participants with the actual rooms with the best match in terms of profile similarity, geographical proximity and random exploration, we first asked them to express their interests. We just provided three simple and very general options: cooking; playing football; gaming. Based on the answer, we provided participants with the appropriate Figma prototype. Basically, we created three Figma prototypes and we presented the user only the one created on the basis of the selected interest.

For the user study, we created three personas, each of them associated to a specific virtual room for better highlighting their personality, interests, and provided services. Our three personas were living in Cesena and were Anna, Matteo, and Luca.

**Anna** has long-time friends that she cares about and meets often and regularly. She loves to share her knowledge about her culture, in particular culinary culture, coming from Emilia Romagna food is very important to her. She is very careful about details and loves quality ingredients. She loves animals and, in particular, her dog, films, and contemporary art. She practices yoga at home and in the nearby studio. Anna loves the sea, so she takes her dog to run on the beach in the winter and, as soon as the summer starts, she loves to go swimming in the sea.

**Matteo** is an architect, and he lives with his girlfriend and beloved dog. He is very extroverted and likes to meet new people. He loves his city and knows a lot about its history and in particular about its historic buildings. I can walk the street of Cesena and talk about it for hours. He walks with his dog, sometimes stops to talk to tourists, helps them find their way, and gives them tips. Sometimes they end up going to a cafe and having coffee while he explains to them all about the wonderful Cesena.

**Luca** is a musician and, to make ends, meet works at the supermarket as a cashier. But as soon as work is done, he goes home and picks up his guitar. He is composing a new Album. He also plays in a band, and they do covers, play at birthday day parties, sometimes in pubs as well. He also loves beer, and he is an expert in the best draft beers pubs in town. When he comes home after playing, he plays games online, often with strangers from the other side of the world.

During the prototype test, we asked the participants to look carefully at all of the three rooms. As a matter of fact, they could see one room as the most similar based on the participant’s interests, one as the nearest, and the last one as the random one. Participants were free to interact with the Figma prototype as long they need.

### Questionnaire

The questionnaire provided after the interaction with the prototype was aimed to analyze the three dimensions we coined in our research framework: profile similarity, geographical proximity, and random exploration, and it was composed of six steps, as shown in Table 1.

**Step 1: questions on locals (Q1-Q6).** We first asked the participants to answer six closed-ended questions to understand if they explored and looked carefully at the rooms. The questions were related to the personas we created and covered their room, hobbies, and services provided. We decided to include this set of questions to test the participant’s attention during the interaction and then apply a quality control check.

**Step 2: questions on the proximity similarity and on the recommendation systems (Q7-Q13).** In the second step, we asked the participants to answer a 5-point Likert scale (from 1-Strongly disagree to 5-Strongly Agree) and an open question about the usefulness of the proximity similarity in the given context (Q7-Q8). Moreover, to better investigate if the recommendation system behind influenced the participants’ opinion, we also asked 4 5-point Likert scale questions and an open question to analyze their trust in them (Q9-Q13). In this case, we were inspired by [43].

**Step 3: questions on the geographical proximity and privacy issues (Q14-Q18).** In the third step, we asked the participants to answer a 5-point Likert scale (from 1-Strongly disagree to 5-Strongly Agree) and an open question about the usefulness of the geographical proximity in the given context (Q14-Q15). Moreover, we investigated if the interest in the “hyper-local tourism” (intended on the very specific area/community/neighborhood) influenced the previous answers through a 5-point Likert scale (Q16). Finally, we investigated the privacy issue, trying to understand if the participants would have preferred to share with the app the GPS position, their interests, both of them or neither of them through a closed-ended question and an open question (Q17-Q18).

**Step 4: questions on the random exploration and serendipity (Q19-Q23).** In the fourth step, we asked the participants to answer 2 5-point Likert scales (from 1-Strongly disagree to 5-Strongly Agree) and an open question about the usefulness of the random exploration in the given context (Q19-Q21). Finally, we asked two 5-point Likert scale questions related to the concept of serendipity linked the random local (Q22-Q23) inspired by [11].

**Step 5: general comments/preferences on the three dimensions (Q24-Q25).** In the fifth step, we asked the participants to answer a closed-ended question and an open question about the preferred criteria to select the local (Q24-Q25).

**Step 6: personal questions (Q26-Q53).** In the sixth step, we asked the participants to answer the Ten Item Personality Measure (TIPI) questionnaire (Q26-Q35) [21] and the Toronto Empathy Questionnaire (TEQ) (Q36-Q51) [45] to gain insight on their personality and empathy’s level, to better investigate if they influenced the previous answers. Moreover, we asked three personal information about the gender and age (Q52-Q53).

**Table 1:** Questions asked in the questionnaire during the user study to analyzed the three dimensions of profile similarity, geographical proximity, and random exploration inside ShareCities

ID	Question
**Q1**	In Anna’s room, which food was on the table?
**Q2**	Which is Anna’s favorite hobby?
**Q3**	Matteo doesn’t like playing football.
**Q4**	Matteo likes provide historical guided tour.
**Q5**	Luca is a wine sommelier.
**Q6**	Luca likes metal music.
**Q7**	I find useful the possibility to visualize the room of
	the local who most match my interests and personality.
**Q8**	Please, explain your previous answer.
**Q9**	I trust recommendation system.
**Q10**	Please, explain your previous answer.
**Q11**	I am willing to let the recommendation system
	help me choose the local who best fit my interests / personality.
**Q12**	I feel secure about relying on the recommendation system
	to choose the local who best fit my interests / personality.
**Q13**	I think the recommendation system knows what I want / what I like.
**Q14**	I find useful the possibility to visualize the room of the local nearest to me.
**Q15**	Please, explain your previous answer.
**Q16**	I think geographical proximity could enhance
	the possibility to better explore “hyperlocal tourism”.
**Q17**	Are you more willing to share your GPS position or your interests with the app?
**Q18**	Please, explain your previous answer.
**Q19**	I find useful the possibility to visualize the room of a random local.
**Q20**	Please, explain your previous answer.
**Q21**	I think random exploration could provide me with the possibility
	to meet diverse people, facilitating unexpected connections among even distant ideas.
**Q22**	The random local was a pleasant surprise.
**Q23**	The random local was unexpected.
**Q24**	What criteria do you think would be most helpful
	in discovering and experiencing authentic travel experiences?
**Q25**	Please, explain your previous answer.
**Q26–Q35**	Ten Item Personality Measure (TIPI) [21]
**Q36–Q51**	Toronto Empathy Questionnaire (TEQ) [45]
**Q52**	To which gender identity do you most identify?
**Q53**	What is your age?

## Results

### User sample

126 participants answered our questionnaires. Considering the background, all of them were students enrolled in Computer Science and Engineering degrees (including Bachelor’s and Master’s degrees). This was due to the methodology exploited to engage them. We invited CS students enrolled at the Web Technologies class (Bachelor’s Degree in Computer Science), and Web services class (Master’s Degree in Computer Science), using the snowball sampling method. Participation was voluntary based on an informed consent. We selected such groups based on the fact that young-adults are our main target audience.

However, based on our attention questions (Q1-Q6), we rejected 20 of them, as they answered in the wrong way to two or more of them (about 16%). Hence, 106 participants passed the quality control check (P1-P106). Adding more details, on a scale between 1 and 7, our participants had low average score in extraversion (*μ* = 3.6, *σ* = 1.3) and agreeableness (*μ* = 4.4, *σ* = 1.0); while they had high average score on conscientiousness (*μ* = 5.2, *σ* = 1.3) and emotional stability (*μ* = 4.4, *σ* = 1.2), and average score on openness to experiences (*μ* = 5.0, *σ* = 1.1) (Q26-Q35). The reference values were taken from [44]. Concerning their empathy level,on a scale between 0 and 64, they scored on average low in the TEQ questionnaire (*μ* = 43.1, *σ* = 7.5) (Q36-Q51). The reference values were taken from [45]. Finally, our participants were 83 male and 17 female (Q52) aged between 18 and 54 years old (Q53).

### Profile similarity

**Fig. 4:**
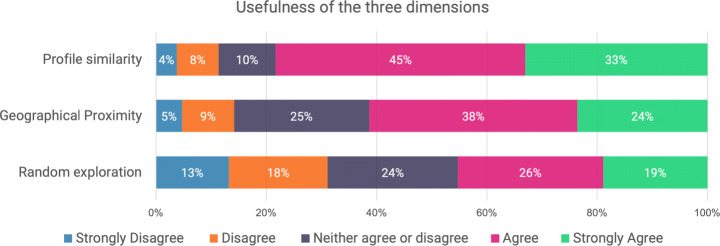
The results for the usefulness of the three criteria: profile similarity (Q7), geographical proximity (Q14), and random exploration (Q19)

Concerning the profile similarity, when we asked our participants if they found useful the possibility to visualize the room of the local who most match their interests and personality through a 5-point Likert scale from 1 to 5, the answers were generally positive (*M* = 4.0, *Mdn* = 4.0, *SD* = 1.3) (Q7), as shown in Fig. 4. This positive approach to profile similarity was also demonstrated by the answers to the open question Q8. For example, P103 stated: “It is important because, if I have to visit a city, I’d like to be guided by a person who gives me similar vibes to mine.”, and P98: “It is useful to have suggestions that match the user’s tastes, which will increase the odds of a successful experience for both parties.”. As a matter of fact, many participants highlighted the advantages of this criteria to look for a local. In particular, it could be useful to find new friends (P3: “Basic interests are important in categorizing users, and is the faster mode for find friends.”), to engage in interesting conversations and discussions (P48: “Usually those with similar interests can provide advice and an interesting debate can arise.”), it can be more engaging, easier, and quick (P101: “When you visit a new town, and you don’t have much time, it is better to be matched a soon as possible with a similar profile to better interact with common interests.”), and can provide new knowledge (P27: “I think that is very useful so you can learn new things about your favorite interest.”.). However, some participants (12 out of 106) considered this similarity a bit limited, hence not fully useful (e.g., P105 stated: “Not really, because I would also like to explore new points of view.”, and P42: “It may be a nice concept, but at the same time I like to meet people with totally different interests.”).

Considering the trust in the recommendation systems, the participants had on average a neutral/slightly positive opinion about them (*M* = 3.7, *Mdn* = 4.0, *SD* = 0.8) (Q9), as shown in Table 2. 44 of the participants recognized the general usefulness (Q10), as reinforced by P14 in the comment “nowadays we are surrounded by these systems”. For example, P95 stated that “Because in a world full of possible choices (e.g., which movie to watch, which product to buy, ...) recommendation system are needed and useful” and P33 wrote that “It makes my life easier”. However, 24 participants acknowledged that the usefulness depend on the system’s algorithm and implementation. As a matter of fact, P53 stated that: “Maybe I trust some recommendation systems and I don’t trust some others.”, or P58: “I don’t fully trust them because they may be subject to bias and/or not consider all relevant parameters to correctly suggest and recommend.”. Moreover, 9 of them perceived a privacy issue when using this type of systems, that affected their evaluation on their trust. For example, P40 stated: “I think profilation is good if helps you have a better experience of the product. Despite that, i think too many companies are using it to sell too many targeted ads and sometimes it can appear scary too.” and P7: “they are usually pretty good there is a privacy issue though”. Finally, 9 of them would trust these systems more if they can also personalize the inputs and the results or have some kind of control on the algorithm. In this scenario, P2 stated: “although a recommendation system can give you good results an user should be able to personalize it’s behavior in the application.”, and P44: “I trust them in what they are doing, but I often like to choose a different option.”. Another problem arose with the recommendation systems was that “In my opinion recommendation systems tend to recommend always the same things, I am a person who likes to explore many different topics” (P38), so their usefulness can decrease with time.

**Table 2:** Percentage for each group for the 5-point Likert scale questions

ID question	Strongly Disagree	Disagree	Neither agree or disagree	Agree	Strongly Agree
Q9	1.9%	2.8%	38.7%	**41.5%**	15.1%
Q11	3.8%	2.8%	27.4%	**46.2%**	19.8%
Q12	4.7%	10.4%	**36.8%**	34%	14.2%
Q13	4.7%	15.1%	**35.8%**	**35.8%**	8.5%
Q16	1.9%	8.5%	22.6%	**50.9%**	16.0%
Q21	0.9%	12.3%	18.9%	**39.6%**	28.3%
Q22	6.6%	14.2%	**34.9%**	27.4%	17.0%
Q23	4.7%	16.0%	**36.8%**	34.0%	8.5%

We tested the correlation between these answers and the ones from Q7 with Spearman correlation. The results showed a very weak correlation between the two variables (*r* = 0.19, *p* = 0.049). Hence, the trust in this type of system doesn’t affect the opinion on the usefulness of the profile similarity.

Considering the willingness to let the recommendation systems help in decision making for finding a local, the participants had on average a neutral/slightly positive opinion about them (*M* = 3.8, *Mdn* = 4.0, *SD* = 0.9) (Q11), as shown in Table 2. We tested the correlation between these answers and the ones from Q7 with Spearman correlation. The results showed a positive moderate correlation between the two variables (*r* = 0.46, *p*< 0.001). Hence, the willingness to let this type of system help choose a local moderate affects the opinion on the usefulness of the profile similarity.

Considering the feeling of security about relying on the recommendation system to choose the local, the participants had on average a neutral opinion about them (*M* = 3.4, *Mdn* = 3.0, *SD* = 1.0) (Q12), as shown in Table 2. We tested the correlation between these answers and the ones from Q7 with Spearman correlation. The results showed a positive weak correlation between the two variables (*r* = 0.39, *p*< 0.001). Hence, the feeling of security weakly affects the opinion on the usefulness of the profile similarity.

Finally, considering the thought that recommendation systems knows what the user wants/likes, the participants had on average a neutral opinion about it (*M* = 3.3, *Mdn* = 3.0, *SD* = 1.0) (Q13), as shown in Table 2. We tested the correlation between these answers and the ones from Q7 with Spearman correlation. The results showed a positive weak correlation between the two variables (*r* = 0.33, *p*< 0.001). Hence, the fact that recommendation systems knows what someone wants/like weakly affects the opinion on the usefulness of the profile similarity.

Moreover we tested the correlation between Q9 and Q13 with Spearman correlation and, as we expected, there was a positive moderate correlation between the two questions (*r* = 0.47, *p* < 0.001).

Summing up, the opinions that our participants had on the usefulness of the profile similarity were weakly or moderately affected by what they thought about the recommendation system that created that similarity.

### Geographical proximity

Concerning the geographical proximity, when we asked our participants if they found useful the possibility to visualize the room of the local nearest to their GPS position through a 5-point Likert scale from 1 to 5, the answers were generally neutral or weakly positive (*M* = 3.7, *Mdn* = 4.0, *SD* = 1.1) (Q14), as shown in Fig. 4. The major flaws found by the participants on this dimension were the fact that “the match with a person is made without looking at common interests.” (P55 on Q15) and “the closer you stay, the higher are the chances of getting a place you already know well.” (P62). However, 13 participants mentioned that proximity could be a different way to meet new people and find new friends. For example, P101 wrote that “it could be a new way to break the ice in meeting people” and P77 mentioned that “it can introduce you to new interests and make immediate connections and outings, shifting your focus less to your interests and more to your city”. The focus on the city and the surroundings appeared in 3 comments. In fact, P100 stated that “It may make me want to explore that area of the city.” and P96: “It could be helpful to encourage exploration of nearby”. Moreover, staying in the surroundings may avoid the risk of getting lost (P98: “Geographical proximity is important when visiting a new place, as it might reduce the probability of the user getting lost and ending up somewhere else.”) and could be quicker and more comfortable, without having to take public transport (P95: “it could be useful to evaluate people who are closer and possibly more comfortable to reach.”). Finally, the proximity could be used for 4 participants as a decision parameter when choosing a local. As a matter of fact, P66 stated that “I believe that the proximity of the room can be in many occasions the discriminating factor between two locals.” and P80 mentioned that: “can be helpful to take better decisions.”.

Considering the thought that geographical proximity could enhance the possibility to better explore “hyperlocal tourism” (tourism based on the very specific area/community/neighborhood), the participants had on average a positive opinion about it (*M* = 3.7, *Mdn* = 4.0, *SD* = 0.9) (Q16), as shown in Table 2. We tested the correlation between these answers and the ones from Q14 with Spearman correlation. The results showed a positive moderate correlation between the two variables (*r* = 0.55, *p*< 0.001). Hence, the possibility to better explore “hyperlocal tourism” affected the opinion on the usefulness of the geographical proximity.

Considering the privacy issue of the data shared with the application, 44 participants (41.5%) preferred to share only their interests, 10 participants (9.4%) were willing to share only the GPS position, 36 (34.0%) both GPS and interests, while the remaining 16 (15.1%) none of them (Q17). This data demonstrated that our participants were aware of the privacy issue behind the mobile applications. However, two participants consider their interests more sensitive information, as mentioned by P66 on Q18: “sometimes sharing interests is too invasive.”. The participants who were more in favor of sharing their interests, stated that “I would have no problem in sharing my interests with the app, but I would be more reticent in sharing my GPS position due to privacy concerns.” (P34) or “I don’t trust to share my position. You never know in the hands of who will end up that data.” (P48). The participants who were willing to share both interests and GPS recognized that these permissions were necessary to have a better experience during the use of the app. As a matter of fact, P5 mentioned that “sharing both of them would let the app give me all that it has to offer.” and P45 stated that: “I think that with GPS position and Interests combo it’s possible to have more accurate results.”. Finally, the participants (12 out of 106) who weren’t willing to share any data usually explained that “data can be stolen” (P31) or “I don’t like sharing any kind of personal information” (P32).

### Random exploration

Concerning the random exploration, when we asked our participants if they found useful the possibility to visualize a random room of a local registered into the app through a 5-point Likert scale from 1 to 5, the answers were generally neutral or weakly positive (*M* = 3.2, *Mdn* = 3.0, *SD* = 1.3) (Q19), as shown in Fig. 4. This outcome on random exploration was also braced by the answers to the open question Q20. Some participants (33 out of 106) would not use this functionality because they didn’t find it useful (as P87 said on Q20: “it is fancy but not so useful” or P80: “It’s important to visualize what is interesting to me in a specific place, not a random local.”) and created a feeling of disorientation (P62: “the fact that I know nothing about the match make me feel a little bit bewildered”.). However, seven of them believed that this was a funny strategy to visualize new information as it was a “fun “mini-game” to do” (P56) and other four thought that “It might add an element of surprise, which I personally like” (P42). The majority of the positive comments (26) were about the opportunity that it could create. As a matter of fact, “It could be a way to explore new and different interests, maybe finding something interesting, in a serendipity way” (P99), “Even if a random local probably does not match my interests very much, checking his/her room can still offer an interesting perspective on the area and the people who live there.” (P100), and “Sometimes we could feel the desire to escape from our comfort zone” (P101).

Considering the thought that random exploration could provide the possibility to meet diverse people, facilitating unexpected connections among even distant ideas, the participants had in average a positive opinion about it (*M* = 3.8, *Mdn* = 4.0, *SD* = 1.0) (Q21), as shown in Table 2. We tested the correlation between these answers and the ones from Q19 with Spearman correlation. The results showed a positive strong correlation between the two variables (*r* = 0.62, *p*< 0.001). Hence, the possibility to meet diverse people affects the opinion on the usefulness of the random exploration.

Considering the thought that the random local was a pleasant surprise, the participants had on average a neutral opinion (*M* = 3.3, *Mdn* = 3.0, *SD* = 1.1) (Q22), as shown in Table 2. We tested the correlation between these answers and the ones from Q19 with Spearman correlation. The results showed a positive strong correlation between the two variables (*r* = 0.61, *p*< 0.001). Hence, the fact that the random local was a pleasant surprise affected the opinion on the usefulness of the random exploration.

Finally, considering the thought that the random local was unexpected, the participants had on average a neutral opinion (*M* = 3.3, *Mdn* = 3.0, *SD* = 1.0) (Q23), as shown in Table 2. We tested the correlation between these answers and the ones from Q19 with Spearman correlation. The results showed a weak correlation between the two variables (*r* = 0.21, *p* = 0.028). Hence, the fact that the random local was unexpected slightly affected the opinion on the usefulness of the random exploration.

### Preference on the recommendation criteria

**Fig. 5:**
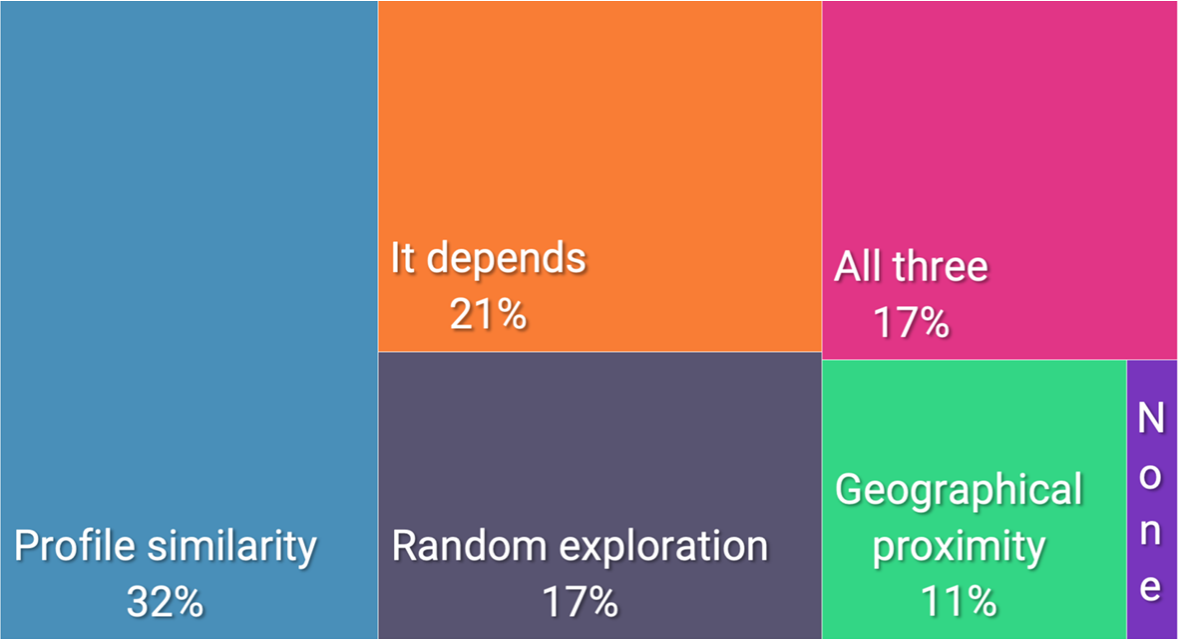
The dimensions preferred by our participants. As demonstrated by the percentage, there wasn’t a clear preference in their choice (Q24)

Concerning Q24, there was no clear preference on the best criteria, as shown in Fig. 5. As a matter of fact, 34 of our participants (32%) preferred the profile similarity, 18 of them (17%) the random exploration, 12 of them (11%) the geographical proximity. However, 18 of them (17%) would like to have all three dimensions, 22 of them (21%) thought that the best dimension depends on the situation, and 2 of them (2%) didn’t like any of the dimensions. To better investigate the different choices, we analyzed the answers to Q25. Regarding the choice of the *profile similarity*, P20 stated that “it is easier to try to interact with people who have tastes and personalities similar to ours”, while P67 recognized the utility of a local guide: “It helps to enjoy a new place based on the things that you like, guided by a point of view of a local.”. The same concept was also expressed by P69: “A local with the same tastes as yours can guide you to places that you are interested in.”. Regarding the choice of the *geographical proximity*, two major topics came up: convenience and budget. P6 stated that “Geographical proximity is more convenient to use.” and P66 added that “Geographical proximity limited the movement around”. However, P5 affirmed that “For economic reasons, maybe, geographical proximity is the best choice”, and the same concept was expressed by P23: “The major target is young people, so I think the Geographical proximity could interest most of them cause their limited budget”. Regarding the choice of *random exploration*, the motivation found was related to the experience of travel. As a matter of fact, P62 stated that: “travel is about experiencing new things, geographical proximity and profile similarity would have the opposite effect in general”, and P84: “I believe that when you travel one of the main objectives is to discover cultures and people and get out of the comfort zone. The criterion that comes closest to this is Random exploration”. The idea of discovery was also present in the comment of P71: “In my opinion during our choices we consider our interests too much and this is right, but sometimes getting carried away by a random choice can help us find new interests that we would have underestimated.”. Regarding the participants who didn’t like any of the criteria, the explanation was the following: “I think travel is an experience that is planned only to the extent of *Where do I land, where do I sleep* the rest must not be pre-written.” (P38). Regarding the choice of all three criteria, 18 participants thought that a combination of all of the three criteria could be an advantage during the exploration of the rooms. In fact, P97 affirmed that: “Random exploration has its importance in making new experiences, but I believe there should be a little bit of familiarity (i.e. profile similarity) in order to make the experience not too random. Also, it could be interesting getting to know the local hidden gems that not many people know about.”, and P78 stated that: “the authentic travel experiences must have all three previous characteristics for be like a real experience”. Finally, regarding the last participants who have chosen the option “It depends”, the motivations were linked to the usefulness of all the three criteria that can be used in different situations and by different people. For example, P13 stated: “I think it changes for each individual and how they relate with new people and places”, P35 added: “It depends on what kind of experience I want to do when the time comes”, and P98: “The importance of the criteria depends on the nature of the travel experience”.

**Fig. 6:**
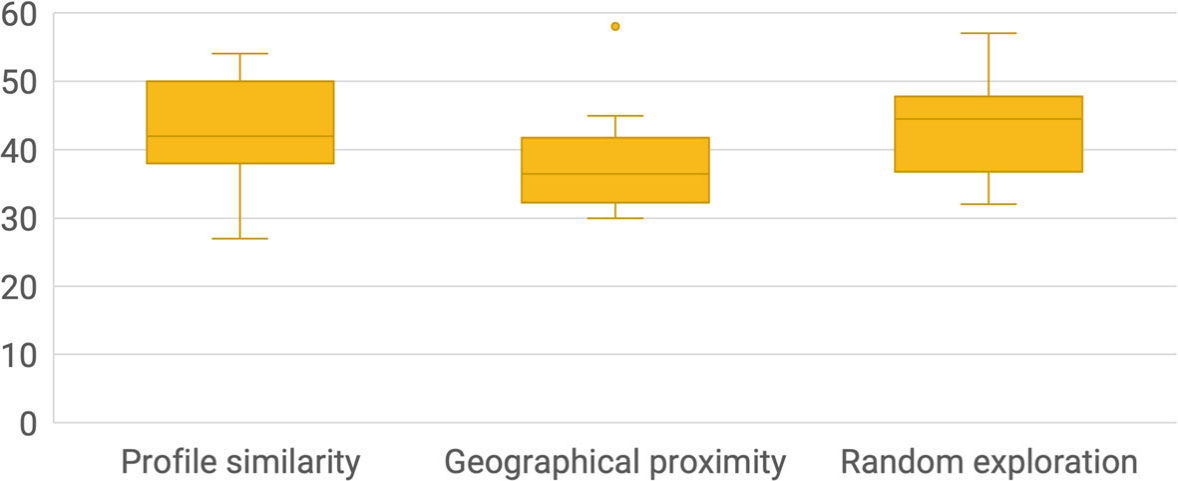
The TEQ scores for the participants divided by the dimension preferred (Q24). The participants who chose the geographical proximity had on average the lowest score in the TEQ

To gain more insight on the study, we decided to investigate the possible relationship between the dimension chosen (profile similarity, geographical proximity and random exploration) and the participants’ profiles. In particular, as shown in Fig. 6, we analyzed the TEQ scores divided by the dimension chosen. From the boxplot, we noticed that the participants who chose the geographical proximity had on average the lowest score in the TEQ. Hence, Kruskal-Wallis Test was conducted to examine the differences on the TEQ score according to the dimension chosen: profile similarity, geographical proximity and random exploration (Q24). The results indicated that there was a significant difference (*H*(2) = 6.031, *p* = 0.049) between the three dimensions, with a mean rank TEQ score of 35.31 for the profile similarity, 20.63 for the geographical proximity, and 35.11 for the random exploration.

**Fig. 7:**
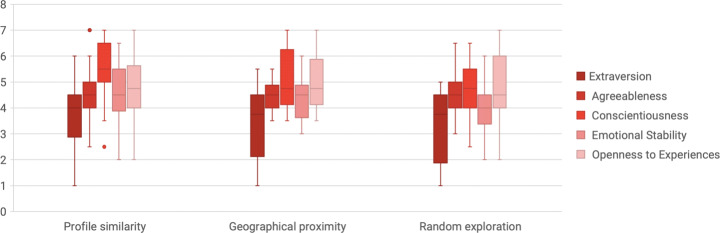
The TIPI score for each traits (extroversion, agreeableness, consciousness, emotional stability, and openness to experience) for the participants divided by the dimension preferred

Moreover, as shown in Fig. 7, we analyzed the TIPI scores for each of the five traits (extroversion, agreeableness, consciousness, emotional stability, and openness to experience) divided by the dimension chosen. From the boxplot, we did not notice any particular difference between the three groups. Hence, we conducted the Kruskal-Wallis Test to examine the differences on the Big-Five personality traits from the TIPI with the dimension chosen. Regarding the extraversion, the results indicated that there was no statistically significant difference (*H*(2) = 1.22, *p* = 0.54) between the three dimensions. Regarding the agreeableness, the results indicated that there was no statistically significant difference (*H*(2) = 0.006, *p* = 0.997) between the three dimensions. Regarding the conscientiousness, the results indicated that there was no statistically significant difference (*H*(2) = 5.87, *p* = 0.053) between the three dimensions. Regarding the emotional stability, the results indicated that there was no statistically significant difference (*H*(2) = 2.89, *p* = 0.24) between the three dimensions. Regarding the openness to experiences, the results indicated that there was no statistically significant difference (*H*(2) = 0.08, *p* = 0.96) between the three dimensions.

## Discussion

After interacting with the prototype of the ShareCities mobile app, our participants were exposed to three different recommendation criteria. The aim was to find out the best criteria to implement in the tourist context to foster the interaction between tourists and locals and to have more probability of living an authentic experience. However, our user study didn’t highlight a preferable criterion; as presented in Fig. 5, a clear majority doesn’t emerge. Our participants generally liked the profile similarity, but the trust in the algorithm influenced their opinion on the usefulness of this criterion. At the same time, a privacy issue emerged in their comments. As a matter of fact, existing literature highlights privacy as one of the main factors that could affect mobile recommendation systems [36, 47]. The privacy concern was also a major flaw of the geographical proximity criterion. Nevertheless, it could be seen as more convenient, in terms of time and money, and a way of experiencing “hyper-local” tourism. Interestingly, this recommendation could also be seen as a way to interact and make new friendships with locals even distant from their interests. For this aspect, it was similar to the random exploration. The participants highlighted both advantages and disadvantages of this criterion. As a negative aspect, the random local could create a feeling of disorientation in some participants. However, for others could be a way of stepping outside their comfort zone and discovering new interests.

This variety of opinions emerged also when analyzing the user’s profile in relation to her/his peculiar point of view. Accordingly, some of the findings and comments from our participants could suggest a link between the personality and the preferred criterion, but, as demonstrated, there was no statistically significant relationship between the criterion and the Big-Five personality traits from the TIPI questionnaire. However, a statistically significant relationship was found between the criterion and the participants’ empathy level, analyzed through the TEQ score.

Finally, the analysis demonstrated that all three criteria could be a good choice in the tourism context, both used alone and in combination, due to the specific scenario and nature of the travel. Moreover, this finding highlighted the necessity of having some kind of personalization strategy to have a better experience within the app and during the actual travel. Indeed, in line with [25], the personalization strategy in the Smart Tourism Technology can affect the touristic experience, but its effect could be moderate by the perceived security/privacy issues.

As a final remark, it is worth noting that, although we provided participants with only three local rooms options (one for each recommendation criterion), we are confident that such a decision didn’t influence our results. We recall that the main objective of this study was to investigate the concept behind the three types of recommendation (named profile similarity, geographical proximity, and random exploration) in terms of the target users’ preferences. Accordingly, in the evaluation study, we asked general questions based on the three concepts that did not concern the satisfaction with the single rooms provided. Hence, we can assume that the results are not influenced by the number of rooms we provided.

### Limitations

The main limitation of this study is the experiment sample, in terms of (i) size, (ii) background, and (iii) nationality.

Size: we were able to engage 126 participants that clearly does not represent a statistically significant sample size if considering the whole population of young-adults as the main target of our system. Future studies should include larger sample sizes to achieve more accurate findings. Nonetheless, analyzing in detail the obtained results, we can affirm that data provide valuable information given the research hypothesis framed in this study, and such insights can be useful for developing cumulative knowledge [29].

Background: the uniform background of the users might produce bias. All the engaged users were enrolled in the Computer Science bachelor’s and master’s degrees. We are aware that the underline condition could have affected our results, in particular when discussing technological issues, such as privacy, trust, and data protection. Despite that, several studies investigated young-adult users’ perception when using the smartphone, enforcing the fact that it is an actual issue that people are aware of [5, 20].

Nationality: all the participants were Italian. Also, in this case, we are confident in thinking that this condition did not strongly affect our study due to the nature of the application. However, to validate our assumptions, a future experiment with a larger number of young-adults with different nationalities should be performed.

## Conclusion

In this paper, we presented an extended analysis on the opportunity to use people-to-people recommendation criteria based on two people (i.e., a local and a tourist) proximity, in terms of profile similarity (investigated both considering the closest profiles and two random ones), and geographical proximity. We hence defined three criteria (that correspond to the three dimensions of interest for our analysis: i) profile similarity, ii) geographical proximity, and iii) random exploration. Through an online questionnaire, we collect answers from 126 young-adults students.

Results highlight a general positive interest in using all the three proximity-based recommendation criteria, while outlining some concerns in terms of privacy and trust (when considering profile similarity), privacy (when considering geographical proximity), and disorientation (when considering random exploration).

For the purpose of this paper, where we wanted to investigate the user’s interest in adopting the three proximity-based recommendation criteria, we computed the profile similarity only based on Jaccard Similarity. However, as future work, we can deeply analyze this similarity, investigating the effectiveness of Jaccard similarity in comparison with other well-known metrics (i.e., Sørensen–Dice coefficient).

As a final remark, it is important to notice that in this paper we focused on the tourists’ point of view. As future work, an investigation focusing on the locals’ point of view is needed [4]. For example, a rewarding system targeting the locals could be a way to improve the overall app experience, making them more willing to share information and interests about themselves and provide services to the tourists. As a matter of fact, previous work had investigated the use of gamification in the tourism context [3, 33]. This aspect could be definitely investigated in future work.

## Footnotes

1 We use the term *hyper-local tourism* to indicate a tourism that is devoted to better know a specific local area or community or neighborhood. This definition was made accordingly with the conceptualization of the hyper-local concept as related to a small community or geographical area (Source: https://www.oxfordlearnersdictionaries.com/definition/english/hyperlocal)

2 https://www.airbnb.it/

3 https://www.couchsurfing.com/

4 https://flutter.dev/

5 https://vuejs.org/

6 https://nodejs.org/

7 https://www.mongodb.com/

8 https://opencv.org/

9 https://pub.dev/packages/panorama

10 https://skillscouter.com/popular-common-hobbies/

11 https://docs.google.com/forms/

12 https://www.figma.com
